# The Readability of Patient-Facing Information in Public Reporting System: Cross-Sectional Evaluation of the City of Helsinki Website

**DOI:** 10.2196/93241

**Published:** 2026-10-08

**Authors:** Lanmei Du, Reima Suomi

**Affiliations:** 1 Department of Management and Entrepreneurship, Turku School of Economics, University of Turku, Rehtorinpellonkatu 3, Turku, 20500, Finland, + 358 417296016

**Keywords:** public reporting, readability, patient education materials, website evaluation, health literacy, health care transparency, digital health

## Abstract

**Background:**

Public reporting websites have become increasingly important for improving health care transparency and supporting informed decision-making. However, little empirical evidence exists regarding whether the readability of these websites adequately meets users’ information needs, particularly in the context of digital health and Finland’s ongoing social welfare and health care reform.

**Objective:**

This study aimed to evaluate the readability of health information on the City of Helsinki public reporting website.

**Methods:**

A cross-sectional website evaluation was conducted using the English-language health care section of the City of Helsinki public reporting website. A total of 429 web pages were collected, among which 246 unique textual descriptions were identified for readability analysis. Website text was automatically extracted using Python and evaluated using 7 established readability indexes: Flesch Reading Ease Score (FRES), Flesch-Kincaid Grade Level (FKGL), Gunning fog index (GFI), Simple Measure of Gobbledygook (SMOG), Coleman-Liau index (CLI), automated readability index (ARI), and Dale-Chall readability score (DCRS). Descriptive statistics were used to summarize textual characteristics and readability outcomes.

**Results:**

All readability indexes consistently demonstrated that the website content exceeded the readability levels recommended for patient education materials. The mean FRES was 42.33 (SD 11.12), indicating difficult-to-read content. The mean FKGL score was 12.03 (SD 2.15), corresponding to a 12th-grade reading level, whereas the mean GFI score of 14.43 (SD 2.43) indicated undergraduate-level reading ability. Similar levels of linguistic complexity were observed across the SMOG (mean 13.69, SD 1.70), CLI (mean 13.61, SD 1.87), ARI (mean 13.67, SD 2.56), and DCRS (mean 11.49, SD 1.02). Considerable variability in readability across web pages suggested inconsistent linguistic accessibility within the website.

**Conclusions:**

The City of Helsinki public reporting website could be improved by enhancing readability and user-centered communication. These improvements may facilitate users’ understanding of health care information and support informed health care decision-making. The findings provide practical implications for the design and evaluation of future digital health care public reporting systems.

## Introduction

With the digitalization of health care, public reporting websites have become an important channel for increasing transparency and supporting informed decision-making [1]. Public reporting provides accessible information about the performance of hospitals, physicians, and health care providers, including treatment outcomes, infection rates, and case complexity [2-5]. Public reporting information enables patients to compare organization providers and may support their choices as part of shared decision-making within patient-physician comanagement [6-9].

However, the usefulness of public reporting depends not only on whether information is publicly available but also on whether users can understand and interpret it correctly [10-13]. Medical information differs from many other types of information because misunderstanding may have direct and potentially irreversible consequences for patients [14,15]. Therefore, health care information should be presented with particular attention to accuracy, clarity, comprehensibility, and unbiased reporting [16-22]. Clear explanations, appropriate contextualization, and understandable presentation of quality indicators can help users with different levels of health literacy interpret public reporting information and avoid misjudgment [23-27].

This issue can also be understood as a problem of representation. As illustrated by Plato’s allegory of the cave, what people perceive may be a representation rather than reality itself [28]. Similarly, developers and users of health care information systems interact with coded and presented representations of health care reality rather than with health care reality directly [29,30]. What patients encounter on a public reporting website is therefore not health care quality itself but a particular representation of that quality through indicators, numbers, labels, explanations, and textual descriptions [18]. From a human-computer interaction perspective, Norman [31] described a “gulf” between users’ conceptualization of a task and the interface presented to them. How information is presented and communicated through an interface may therefore influence users’ ability to understand and use it [32,33].

Information quality is therefore an important consideration in the design and evaluation of public reporting websites [34-36]. Information is generally understood as processed data that are meaningful and valuable for current or future actions and decisions [37]. Higher information quality has been associated with greater user satisfaction and more effective system use [38]. In particular, the representational quality of information concerns how information is presented to users, including its simplicity, presentation style, and comprehensibility [39]. For public reporting websites, these characteristics are especially important because quality indicators and other performance measures may be difficult for patients to interpret when they are highly technical or insufficiently explained [40-44]. In addition, narrative or other unstructured language may require greater processing effort, providing context that can supplement and support the interpretation of data-driven information [45,46].

One important aspect of information presentation is readability. Readability affects the effort required to process written information and may influence whether users can successfully obtain and understand the information they need [47]. Although previous research has examined public reporting in relation to patient choice, patient satisfaction, transparency, and health care quality [48-52], empirical research assessing the actual medical information presented on public reporting websites remains limited [53-56]. In particular, little is known about whether patient-facing public reporting information is sufficiently readable to support users’ information needs in the context of digital health and shared decision-making.

This issue is particularly relevant in Finland, where the social welfare and health care reform emphasizes freedom of choice and the development of public reporting systems to support informed choices [57]. As public reporting systems and digital health services become increasingly important sources of health care information, evaluating the readability of patient-facing public reporting content can help determine whether these systems effectively communicate information to the public [58-60]. Therefore, this study asked the following question: How readable is patient-facing public reporting information? To address this question, we evaluated the readability of the City of Helsinki public reporting website.

## Methods

### Study Design

This study used a cross-sectional website evaluation design. A total of 429 web pages were collected from the health care section of the City of Helsinki website at the page level. Accordingly, the analysis was restricted to the health care section of the City of Helsinki website, which was selected as the primary object of analysis. Representative screenshots of the City of Helsinki health care website are provided in Figure 1 to illustrate the website interface and page layout.

**Figure 1.**
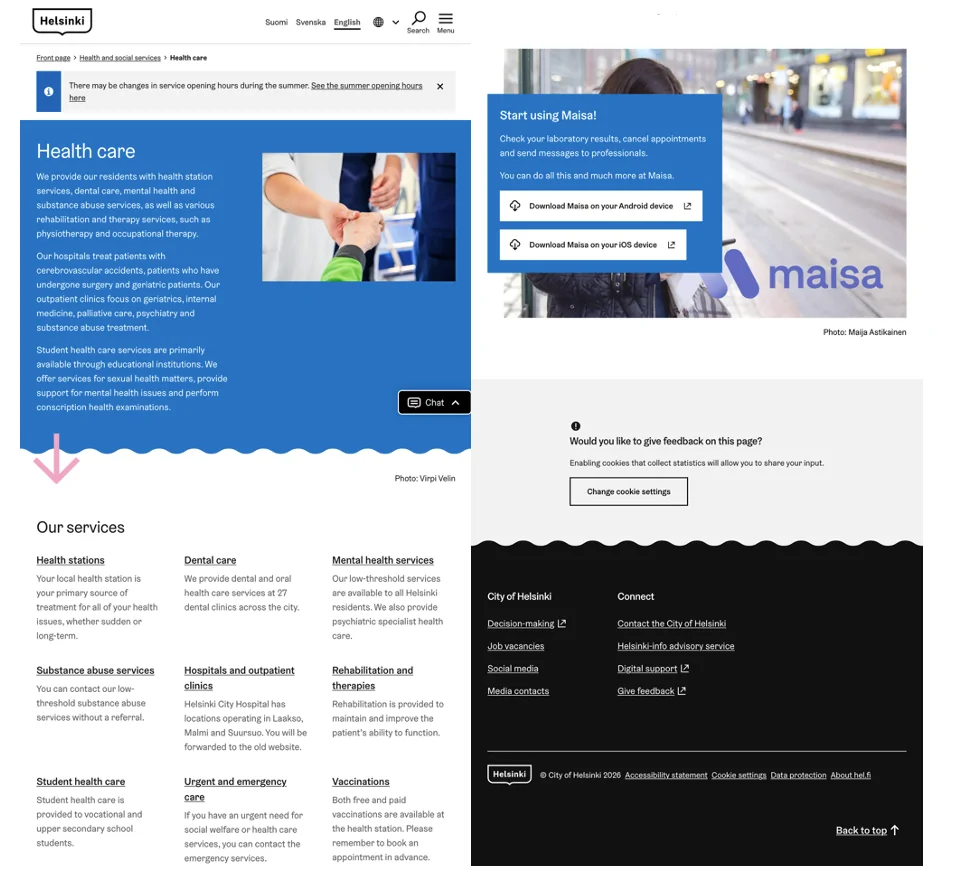
Home page of the health care section of the City of Helsinki website as an example of a health care website, presented in 2 consecutive sections because of the web page length.

In this study, public reporting websites were conceptualized as textual information resources. It should be noted that a substantial proportion of the City of Helsinki web pages consisted of short headings and hyperlink labels.

Internal web pages within the City of Helsinki health care website were included in the analysis. Hyperlink labels appearing on these web pages were retained as part of the visible text because they constitute information presented to users. In contrast, hyperlinks directing users to external websites were recorded but not followed or included as analytical units.

Furthermore, because readers do not naturally filter out redundant or navigational content when accessing information on web pages, all textual elements were retained for analysis. Therefore, no minimum text length threshold (eg, excluding texts containing fewer than 100 words) was applied, allowing all visible English-language textual content to be included in the readability analysis.

All eligible visible textual content within each web page was aggregated into a single web page–level text corpus before readability assessment. Therefore, headings and hyperlink labels were not evaluated as independent short text units. Readability was then assessed at the web page level using 7 established readability indexes.

This study is reported in accordance with the Strengthening the Reporting of Observational Studies in Epidemiology (STROBE) guidelines for cross-sectional studies. The completed STROBE checklist is provided in Checklist 1.

### Data Collection

All websites were assessed in November 2025. Data collection was conducted using Python (version 3.9.6; Python Software Foundation), with packages including *Requests*, *Beautiful Soup*, *Fake-useragent*, *urllib.parse*, and *openpyxl*.

First, all textual content was scraped from the website. To ensure accuracy and minimize errors, author LD reviewed the dataset. The study then established a Microsoft Excel sheet containing the URLs of each website (Multimedia Appendix 1).

Subsequently, all 429 extracted URLs, together with the textual content from each web page, were exported to a Microsoft Excel file. The web page was defined as the primary unit of sampling. Subsequent verification of the URLs revealed that some represented anchor URLs pointing to different sections within the same web page. Duplicate textual descriptions across different numbers were identified, revealing that 183 URLs contained duplicated text (Multimedia Appendix 2).

Nevertheless, website users encounter these web pages individually during navigation and do not manually remove duplicate content. After identifying duplicated textual content and identical readability scores, 246 unique textual records were retained for statistical analysis.

The text associated with each URL was recorded as the corresponding description. Only English-language web pages were included because the readability indexes used in this study were validated primarily for English-language text and rely on English-specific linguistic features such as word length, syllable count, and familiar word lists. In addition, PDF documents, images, and other nontextual content (eg, external links) were excluded from the readability analysis.

### Readability Assessment

Following data extraction, web page records with identical textual content and identical readability scores were identified and deduplicated using a custom Python script (Multimedia Appendix 3). Readability indexes and text characteristics were then summarized using descriptive statistics.

Readability-related text characteristics were extracted for each web page, including the total number of characters, words, and sentences; mean sentence length (words per sentence); total syllables; number of polysyllabic words; and the proportion of polysyllabic words among all words (Multimedia Appendix 4).

Readability is commonly defined as the ease with which a text can be read and understood [61]. In health literacy and patient education research, it is often described more precisely as “the study of matching reader and text,” so that the reading level of the material corresponds to the reader’s ability to read with understanding [62].

Readability was assessed as an indicator of linguistic complexity that may influence users’ cognitive processing of public reporting information. Existing conceptual frameworks for public reporting websites include readability alongside dimensions such as accuracy, completeness, design, usability, and accessibility [63]. Automated website assessment frameworks operationalize these dimensions across domains such as accessibility, content, marketing, and technology [64].

Numerical scores were generated automatically using predefined computational algorithms. No subjective human scoring was involved. Before the readability assessment, web page text was preprocessed by removing noncontent elements, including scripts, HTML markup, stylesheets, content from external websites, and PDF documents where applicable.

Within the Nibbler website assessment framework, readability is evaluated as an indicator within the “Accessibility” and “Content” categories [65-67]. Given that the conceptualization and operationalization of readability vary across disciplines, this study used 7 established readability measures to capture complementary aspects of text difficulty. These measures differ in their underlying linguistic features and weighting schemes, allowing the consistency of readability assessments to be examined across alternative operationalizations.

The primary outcomes were the 7 readability indexes (FRES, FKGL, GFI, SMOG, CLI, ARI, and DCRS). Descriptive statistics, including the mean, SD, and minimum and maximum values, were then calculated and are presented in the Results section.

Using the readability measures as shown in Table 1, readability was assessed using the Python Textstat library, which was used to calculate 7 established readability measures: the Flesch Reading Ease Score (FRES) [68], Flesch-Kincaid Grade Level (FKGL) [65], Gunning fog index (GFI) [69,70], Simple Measure of Gobbledygook (SMOG) [41], Coleman-Liau index (CLI) [71], automated readability index (ARI) [72], and Dale-Chall readability score (DCRS) [73]. These indexes estimate the reading difficulty of health-related materials and have been extensively applied in previous studies evaluating online health information [65].

**Table 1.** Standard formulas and descriptions of the readability measures used in this study.

Readability measures	Focus	Scoring basis	Formula
FRES^a^	Scores range from 0 to 100, with higher scores indicating easier readability.	Sentence length; syllables per word	206.835 − (84.6 × ASW^b^) − (1.015 × ASL^c^)
FKGL^d^	Estimates the approximate US grade level required to understand a text, with higher scores indicating greater reading difficulty.	Sentence length; syllables per word	(0.39 × ASL) + (11.8 × ASW) − 15.59
GFI^e^	Estimates the years of formal education required to understand a text, emphasizing sentence length and complex words.	Sentence length; complex words	0.4 × (ASL + [(C^f^/W^g^) × 100])
SMOG^h^	Estimates the approximate grade level required to understand a text based on the number of PWs^i^.	PWs; sentence count	1.0430 × √PW + 3.1291
CLI^j^	Estimates the approximate grade level required to comprehend a text using characters and sentence-based measures.	Character-based word length and sentence-based features	(0.0588 × L^k^) − (0.296 × S^l^) − 15.8
ARI^m^	Estimates the approximate US grade level required to understand a text using character length and sentence length rather than syllable counts.	Sentence length; characters per word	4.71 × (characters/words) + (0.5 × ASL) − 21.43
DCRS^n^	Estimates reading difficulty based on sentence length and the proportion of words not included in a predefined list of familiar words.	Sentence length; DWs^o^	0.1579 × (DW/W × 100) + (0.0496 × ASL) + 3.6365^p^

a FRES: Flesch Reading Ease Score.

b ASW: average syllables per word.

c ASL: average sentence length (number of words per sentence).

d FKGL: Flesch-Kincaid Grade Level.

e GFI: Gunning fog index.

f C: number of complex words after excluding proper nouns, words made polysyllabic by adding “ed” or “es,” and compound words composed of simpler words.

g W: total number of words.

h SMOG: Simple Measure of Gobbledygook.

i PW: polysyllabic word (words with ≥3 syllables).

j CLI: Coleman-Liau index.

k L: average number of letters per 100 words.

l S: average number of sentences per 100 words.

m ARI: automated readability index.

n DCRS: Dale-Chall readability score.

o DW: difficult word not included in the Dale-Chall familiar word list.

p The constant 3.6365 is added when the proportion of difficult words exceeds 5%.

Although some measures rely on overlapping variables, they differ in how these variables are weighted and interpreted. The FRES estimates reading ease, whereas the FKGL, GFI, SMOG, and CLI provide approximate US grade-level estimates. The ARI similarly provides a grade-level estimate based on character and sentence length, whereas the DCRS incorporates lexical familiarity through a predefined familiar word list. Thus, the 7 measures were treated as complementary indicators of readability rather than as 7 independent outcomes.

For interpretation, higher FRES scores indicate greater reading ease, whereas higher scores on the other grade-level or difficulty-oriented measures indicate greater reading difficulty. The US Department of Health and Human Services sixth-grade reading level recommendation corresponds to a FRES score of 80 or higher and FKGL, GFI, SMOG, and CLI scores of 6.9 or lower [74-76].

### Ethical Considerations

This study analyzed only publicly available website content and did not involve human participants, personal data, or any intervention. Therefore, formal ethics approval was not required. According to the Finnish National Board on Research Integrity guidelines, ethical review is generally not required for research based solely on publicly available data [77].

## Results

A total of 246 unique textual records from the health care section of the City of Helsinki website were included in the analysis. Table 2 shows the descriptive statistics of the readability measures for patient-facing website content, whereas the descriptive statistics of the textual characteristics of the website content are provided in Multimedia Appendix 4.

**Table 2.** Descriptive statistics of the readability measures for patient-facing website content.^a^

Readability measures	Mean (SD)	Range
Flesch Reading Ease Score	42.33 (11.12)	5.53-63.13
Flesch-Kincaid Grade Level	12.03 (2.15)	8.41-17.99
Gunning fog index	14.43 (2.43)	10.25-21.75
Simple Measure of Gobbledygook	13.69 (1.70)	10.54-19.05
Coleman-Liau index	13.61 (1.87)	9.60-19.84
Automated readability index	13.67 (2.56)	9.21-22.07
Dale-Chall readability score	11.49 (1.02)	8.85-14.43

a Higher values for the Flesch Reading Ease Score indicate easier-to-read content, whereas higher values for the Flesch-Kincaid Grade Level, Gunning fog index, Simple Measure of Gobbledygook, Coleman-Liau index, automated readability index, and Dale-Chall readability score indicate more difficult-to-read content.

The mean FRES score was 42.33 (SD 11.12), corresponding to a “difficult” readability level according to the established Flesch scale [68]. The mean FKGL score was 12.03 (SD 2.15), corresponding to approximately a 12th-grade reading level and substantially exceeding the recommended range of 6 to 8 [78]. Similarly, the mean GFI, SMOG, CLI, and ARI scores were 14.43 (SD 2.43), 13.69 (SD 1.70), 13.61 (SD 1.87), and 13.67 (SD 2.56), respectively, indicating reading levels substantially above the recommended range. The mean DCRS was 11.49 (SD 1.02), also indicating considerable reading difficulty. Overall, all 7 readability measures consistently indicated that the patient-facing public reporting web pages were written at relatively high reading difficulty levels.

## Discussion

### Principal Findings

This study found that patient-facing public reporting web pages consistently exceeded the readability levels recommended for patient education materials, suggesting that transparency alone does not guarantee that health care information is understandable or usable by patients. Although public reporting websites make health care performance information publicly available, the linguistic complexity of the content indicates a potential challenge for patient-facing communication. This distinction is particularly important when patients seek public reporting information to compare health care providers, understand health care performance, or inform their health care decisions. When information is publicly available but difficult to understand, formal transparency may not translate into meaningful access or effective use.

The magnitude of the observed readability difficulty further underscores this concern. The mean GFI score of 14.43 corresponds to a readability level estimated at approximately 14 years of formal education, or a college-level reading requirement, whereas the SD of 2.43 indicates moderate variation in readability across web pages [79]. The FRES results similarly indicated a college-level reading requirement. These findings substantially exceed the grade 6 to 8 readability level recommended by the American Medical Association and the National Institutes of Health for written health materials, particularly those intended for individuals with limited health literacy [80,81]. Thus, when patients turn to public reporting websites to obtain information relevant to their health care choices, the relatively high reading difficulty observed in this study may represent a potential challenge for comprehension and subsequent use of the information [82]. Rather than simply increasing the amount of information available to patients, patient-centered public reporting should therefore consider whether users can understand and meaningfully apply the information provided.

The consistency of the findings across the 7 readability measures further strengthens this interpretation. Although these measures differ in their underlying variables, weighting schemes, and interpretations, they consistently indicated a relatively high level of reading difficulty. FRES and FKGL both incorporate sentence length and syllables per word but differ in how their scores are interpreted, whereas the GFI emphasizes complex words, the SMOG focuses on polysyllabic words, the CLI and ARI rely on character-based features, and the DCRS incorporates lexical familiarity. The convergence of these measures suggests that the observed difficulty is not attributable to a single readability formula but reflects a broader pattern of linguistic complexity in patient-facing public reporting content. The relatively high GFI scores indicate substantial linguistic complexity across many web pages, potentially including specialized medical terminology that may be difficult for lay users, such as patients and family members, to process [82]. Despite differences in their underlying formulas and weighting schemes, the 7 indexes therefore provide converging evidence supporting the robustness of the overall findings.

These findings also have implications beyond the immediate readability of patient-facing materials. Health literacy involves individuals’ ability to access, understand, evaluate, and use health information, whereas data literacy encompasses the ability to interpret and meaningfully use data in a given context [83]. In increasingly digital health environments, effective use of public reporting information may require both forms of literacy. Within public reporting systems, this process involves multiple groups, including health care professionals who generate or interpret performance information, developers who translate underlying data into digital interfaces, and patients and other users who ultimately interpret and apply the information presented to them. Public reporting websites therefore function not merely as channels for patient education but also as shared information environments in which health literacy and data literacy are enacted. Ensuring that complex performance information is communicated in accessible language may consequently support more equitable participation in the interpretation and use of health care data.

High-quality information is valuable only when it can be readily understood and meaningfully used by individuals with different levels of health literacy [84]. There is previous evidence suggesting that individuals with higher educational attainment tend to prefer access to raw data, whereas those with lower educational attainment rely more heavily on intuitive representations, such as color coding and graphical displays [85-87]. The present study did not examine differences in users’ educational backgrounds or health literacy. Instead, the high readability difficulty observed in the public reporting web pages draws attention to the linguistic accessibility of patient-facing information and the need to consider variation in users’ information needs and capabilities when designing and communicating public reporting information. Future research could examine whether combining appropriately simplified textual information with visualizations and other user-oriented presentation formats can improve users’ comprehension, interpretation, and use of public reporting information.

Previous studies have emphasized that narrative-based communication may strengthen users’ understanding and interpretation of public reporting systems by improving the transparency, credibility, and perceived relevance of publicly reported health care information [88-90]. Building on this literature, our findings suggest that contextualized communication alone may not be sufficient if the language used to convey the information remains difficult for the intended audience to understand. Readability should therefore be considered complementary to narrative and other communication strategies aimed at improving transparency and credibility. In the context of patient-provider shared decision-making, public reporting systems may be more effective when information is not only transparent and contextually explained but also presented in a language that patients can readily understand and apply to their health care decisions.

### Policy Implications for Patient-Centered Public Reporting Information

Increasing transparency of health care performance reporting should be regarded not only as making health care performance data publicly available but also as ensuring that these data are presented in ways that facilitate public understanding. Accordingly, government agencies responsible for public reporting websites may consider incorporating readability evaluation into routine website quality assessment. Automated readability assessment tools could be integrated into website maintenance workflows to identify pages requiring revision and improve the consistency of patient-facing information.

These considerations may become applicable as digital health information is incorporated into AI-enabled health information services. Publicly available web pages may serve as information sources for systems that retrieve, summarize, or generate health-related information. Improving the quality, consistency, and readability of public reporting web pages may therefore have implications beyond direct patient use by improving the quality of information available to downstream digital and AI-enabled health information services. However, this potential benefit should be interpreted cautiously as further research is needed to determine whether improvements in web page readability translate into greater accuracy, interpretability, or reliability of AI-generated health information.

### Practical Implications

Improving the quality of public reporting websites should be considered alongside the broader processes through which health care information is stored, structured, validated, and published. The public reporting data presented on websites represent only a subset of the information maintained within health care databases [91,92]. Website data generally consist of structured and unstructured content, with semistructured formats such as XML functioning as an intermediary layer for data validation, exchange, and standardized representation [93]. Optimizing XML-based data structures during database development can improve the accuracy and consistency of public reporting data, thereby enhancing transparency [94].

At the same time, redevelopment of existing information systems typically requires substantial time, financial investment, and organizational resources, making ecosystem redesign impractical in many health care settings [95]. Under these constraints, optimizing intermediate data structures (eg, XML) represents a practical strategy for improving key data processing workflows and enhancing the consistency and standardization of publicly reported information, thereby supporting more transparent and reliable public reporting. However, improvements in the underlying data infrastructure should complement rather than replace efforts to improve the readability and user-centered presentation of information. Ultimately, effective public reporting requires both reliable underlying data and communication practices that enable patients, health care professionals, and other users to understand and meaningfully use those data.

Previous research suggests that the way in which information is structured and visually presented can influence how users attend to and respond to publicly reported health care information [96-100]. Websites commonly use visual elements such as icons, symbols, and color to communicate information, whereas the quantity and presentation of information may also affect users’ ability to process and use it [101-103]. Studies of public reporting and health care information presentation have further examined users’ responses to quality ratings, visual displays, and other forms of information presentation [96-98]. These findings highlight the importance of considering not only what information is presented but also how it is structured and communicated to users. We provide an initial examination of the readability of the underlying information presented on patient-facing public reporting web pages, highlighting a potential issue at the data and content level that warrants further investigation.

From a practical perspective, data development processes could therefore incorporate annotations and contextual information within the underlying code to better accommodate users. Such annotations could provide greater clarity regarding the meaning and interpretation of data elements, which may contribute to more readable and understandable information when the data are subsequently presented on web pages. Similarly, web page design should consider how colors, symbols, and other visual elements are likely to be interpreted by users. Selecting and applying these elements in ways that are intuitive and readily interpretable may further support the accessibility and usability of patient-facing public reporting information.

### Limitations

This study has several limitations. First, the readability analysis was performed only on web pages with textual content. Although no minimum text length threshold was applied, manual inspection indicated that web pages containing fewer than 100 words were mainly composed of headings, navigation labels, short notices, or introductions. Therefore, these short text segments were retained for descriptive statistical analysis. Future website research should perform word segmentation and apply appropriate text exclusion criteria once sufficient textual data are available to ensure more robust readability assessment.

Second, the readability results were based on automated algorithmic scores rather than direct user testing. During the manual review of all extracted texts, we observed that the public reporting system presented relatively limited performance data but included a dedicated feedback form for reporting website-related issues. As user heterogeneity in health literacy, educational level, language proficiency, and familiarity with health care terminology exists [42,104], future studies could investigate dashboard-based public reporting systems with user testing that integrate audiovisual elements to enhance information presentation [105,106].

Finally, the data were obtained from the English-language health-related pages of a single municipal website in Finland. Therefore, the findings primarily reflect this specific website and its linguistic and operational context, and caution is warranted when transferring the findings to other public reporting environments. Future research could examine public reporting websites across different organizational, operational, and language settings. In particular, comparative analyses of Finnish- and English-language content could determine whether readability differs across language versions of the same website.

### Conclusions

This study evaluated the readability of the City of Helsinki public reporting website from the perspective of health care information design. The findings consistently demonstrated that the website content exceeded the readability levels recommended for patient education materials and exhibited considerable variability across web pages.

These findings suggest that the effectiveness of public reporting depends not only on the transparency of health care data but also on how such information is encoded, organized, and communicated to end users.

Improving readability and narrative-based communication may enhance users’ understanding of public reporting information and, ultimately, promote more informed health care decision-making. Future research should further examine multilingual public reporting websites and investigate how different information presentation strategies influence users’ comprehension and engagement across diverse health care contexts.

## Supplementary material

10.2196/93241 Multimedia Appendix 1 URLs with description.

10.2196/93241 Multimedia Appendix 2 Readability results before deduplication.

10.2196/93241 Multimedia Appendix 3 Readability results after deduplication.

10.2196/93241 Multimedia Appendix 4 Descriptive statistics of the linguistic characteristics of the website content.

10.2196/93241 Checklist 1 STROBE checklist.

## Abbreviations

ARI: automated readability index
CLI: Coleman-Liau index
DCRS: Dale-Chall readability score
FKGL: Flesch-Kincaid Grade Level
FRES: Flesch Reading Ease Score
GFI: Gunning fog index
SMOG: Simple Measure of Gobbledygook
STROBE: Strengthening the Reporting of Observational Studies in Epidemiology
