# Supplementary material for: The Readability of Patient-Facing Information in Public Reporting System: Cross-Sectional Evaluation of the City of Helsinki Website
Source: Online J Public Health Inform. 2026 Oct 8;18:e93241. doi: 10.2196/93241 (PMC13648947; doi:10.2196/93241)
Supplement: Supplementary file 1 [file ojphi-v18-e93241-s004.docx]

**Multimedia Appendix 4. Descriptive Statistics of Linguistic Characteristics of Website Content**

| Category | Mean (SE) | SD | Minimum | Maximum |
| --- | --- | --- | --- | --- |
| Characters | 3562.63 (182.04) | 2855.25 | 686 | 26862 |
| Words | 552.83 (29.36) | 460.43 | 97 | 4344 |
| Average Sentence Length | 18.83 (0.23) | 3.67 | 11.59 | 32.56 |
| Average Word Length | 5.52 (0.19) | 0.30 | 4.82 | 6.57 |
| Syllables | 932.93 (47.29) | 741.67 | 193 | 7132 |
| Average Syllables per Word | 1.72 (0.01) | 0.12 | 1.50 | 2.19 |
| Polysyllabic Words | 97.05 (4.87) | 76.4 | 22 | 774 |
| Complex Word Ratio | 0.18 (0.00) | 0.04 | 0.11 | 0.35 |
